# Supplementary material for: Challenges of BTV-Group Specific Serology Testing: No One Test Fits All
Source: Viruses. 2024 Nov 21;16(12):1810. doi: 10.3390/v16121810 (PMC11680153; doi:10.3390/v16121810)
Supplement: Supplementary file 1 [file viruses-16-01810-s001.zip › viruses-3194725-supplementary.pdf]

Supplementary materials

Sample #	Agent	Species
1	Belmont SH264 S13361 3/6/82	Ovine
2	Bovine Ephemeral Fever 887721 B131 S14019	Bovine
3	Bunyp Creek cs58 r721 s12223	Bovine
4	Corriparta M96 S12162 18/7/79	Ovine
5	Corriparta N99 MRM1 S12661	Ovine
6	CSIRO Village cs11 r838 s12408	Bovine
7	Eubanangee R722 S12196 (In1074) 12/9/79	Bovine
8	Eubenangee A823 S12380 (CSIRO23) 18/12/79	Bovine
9	Eubenangee R956 S12526 (CSIRO32) 16/4/80	Bovine
10	Facey's paddock SH247 S13357 3/6/82	Ovine
11	Kawanyama SH293 S13374 3/6/82	Ovine
12	Koongol SG551 S13359 3/6/82	Ovine
13	Malignant Catarrhal Fever 334 S150976/9/91	Bovine
14	Maputta SH226 S13358 3/6/82	Ovine
15	Marrakai CS82 R853 S12772	Ovine
16	Palyam D'Aguillar A842 S12752 21/7/80	Bovine
17	Palyam D'aguillar b8112 r768 s12231	Ovine
18	Palyam D'Aguillar R878 S12751 21/7/80	Bovine
19	Paroo River SG680 S13356 3/6/82	Ovine
20	Taggert SG554 S13360 3/6/82	Ovine
21	Tilligerry R714 S12230 (NB7080) 8/10/79	Bovine
22	Tilligerry R723 S12230 (NB7080) 8/10/79	Bovine
23	Tilligerry R726 S12230 (NB7080) 9/10/79	Bovine
24	Wallal 022 s12701 (CSIRO44) 2/7/80	Ovine
25	Wallal L28 S12,162 (CSIRO44) 18/7/79	Bovine
26	Warrego N85 S12409 (CSIRO12) 9/1/80	Ovine
27	Warrego N98 S12409 (CSIRO12) 9/1/80	Ovine
28	Warrego R833 S12409 (CSIRO12) 9/1/80	Bovine
29	Warrego R843 S12409 (CSIRO12) 9/1/80	Bovine

Figure S1: Identity of 21 orbivirus and eight other non-orbivirus arbovirus positive sera.

		AAHL C-ELISA		sELISA		IDEXX		VMRD		IDVet	
		Positive ≥32%		>0.72 Positive		<70 %Positive		≥60% Positive		<40% Positive	
Number	EHD Positive panel [1]	% inhib	Interpret	S/P ratio	Interpret	S/N %	Interpret	% inhib	Interpret	% Inhib	Interpret
1	anti EHD 2	1	Negative	2.52	Positive	98	Negative	34	Negative	89	Negative
2	anti EHD 5	64	Positive	1.63	Positive	46	Positive	80	Positive	95	Negative
3	anti EHD 6	16	Negative	0.79	Positive	82	Negative	31	Negative	95	Negative
4	anti EHD 7	-3	Negative	11.03	Positive	100	Negative	13	Negative	94	Negative
5	anti EHD 8	44	Suspect	4.59	Positive	56	Positive	64	Positive	59	Negative
6	12-01347-0007	104	Positive	27.37	Positive	11	Positive	78	Positive	78	Positive
7	12-01347-0008	108	Positive	15.62	Positive	12	Positive	92	Positive	26	Negative
8	06-03694-0001	99	Positive	4.87	Positive	32	Positive	8	Negative	7.16	Positive
9	06-03694-0002	105	Positive	5.82	Positive	5	Positive	-2	Negative	4.48	Positive
10	06-03694-0003	58	Positive	1.74	Positive	42	Positive	1	Negative	38	Positive
11	06-03694-0004	79	Positive	2.84	Positive	40	Positive	77	Positive	28	Positive
12	06-03694-0005	93	Positive	5.5	Positive	14	Positive	74	Positive	6	Positive
13	06-03694-0006	13	Negative	0.88	Positive	80	Negative	31	Negative	115	Negative
14	06-03694-0007	2	Negative	2.64	Positive	97	Negative	13	Negative	96	Negative
15	09-04228-0007	73	Positive	0.17	Negative	94	Negative	95	Positive	86	Negative
16	13-00995-0009	-10	Negative	0.23	Negative	110	Negative	60	Positive	96	Negative
17	13-00995-0003	8	Negative	1.06	Positive	108	Negative	-8	Negative	89	Negative
18	16-00269-0001	-18	Negative	0.13	Negative	125	Negative	-1	Negative	108	Negative
19	15-03524-0001	-15	Negative	-10	Negative	122	Negative	1	Negative	118	Negative
20	16-00126-0001	-16	Negative	-0.13	Negative	150	Negative	13	Negative	118	Negative
21	14-02495-0001	66	Positive	1.14	Positive	64	Positive	39	Negative	56	Negative
22	17-03205-0001	105	Positive	18.15	Positive	11	Positive	15	Negative	7	Positive
23	17-05607-0001	28	Negative	1.31	Positive	113	Negative	5	Negative	5	Positive
24	18-02706-0003	-21	Negative	-0.21	Negative	115	Negative	27	Negative	116	Negative
25	18-02444-0002	-19	Negative	-0.77	Negative	131	Negative	-2	Negative	117	Negative
26	18-03208-0002	-11	Negative	0.47	Negative	94	Negative	-38	Negative	114	Negative

Figure S2: Analytical specificity: EHD positive sera.

			AAHL cELISA		sELISA		IDEXX ELISA		VMRD ELISA		IDVet ELISA	
			≥40 %		≥0.62 or ≥0.36		≤ 70% Pos >79 Neg		≥60%		<50%	
Sample #	Genus	Virus ID	% Inhib	Interp.	S/P ratio	Interp.	% of pos	Interp.	% Inhib	Interp.	% Inhib	Interp.
1	Orbivirus	Eubenangee	-6	Negative	0.81	Positive	102	Negative	24	Negative	78	Negative
2	Orbivirus	Eubenangee	83	Positive	0.31	negative	27	Positive	20	Negative	18	Positive
3	Orbivirus	Eubenangee	-17	Negative	1.42	Positive	92	Negative	35	Negative	111	Negative
4	Orbivirus	Tilligerry	-13	Negative	0.29	negative	76	Suspect	28	Negative	85	Negative
5	Orbivirus	Tilligerry	-15	Negative	0.09	negative	96	Negative	43	Negative	76	Negative
6	Orbivirus	Tilligerry	12	Negative	0.01	negative	116	Negative	22	Negative	147	Negative
7	Orbivirus	Wallal	38	Negative	0.14	negative	121	Negative	48	Negative	133	Negative
8	Orbivirus	Warrego	95	Positive	0.31	negative	13	Positive	72	Positive	7	Positive
9	Orbivirus	Warrego	96	Positive	2.24	Positive	8	Positive	89	Positive	97	Negative
10	Orbivirus	D'Aguillar	7	Negative	0.24	negative	104	Negative	43	Negative	124	Negative
11	Orbivirus	D'Aguillar	-5	Negative	0.32	negative	104	Negative	41	Negative	148	Negative
12	Orbivirus	Bunyp Creek Village	-4	Negative	-0.16	negative	117	Negative	5	Negative	110	Negative
13	Orbivirus	CSIRO Village	79	Positive	0.05	negative	27	Positive	81	Positive	13	Positive
14	Orbivirus	Marrakai	74	Positive	1.58	Positive	30	Positive	85	Positive	98	Negative
15	Orbivirus	D'Aguillar	60	Positive	0.17	negative	45	Positive	69	Positive	32	Positive
16	Orbivirus	Corriparta	-43	Negative	0	negative	90	Negative	8	Negative	124	Negative
17	Orbivirus	Wallal	-16	Negative	0.03	negative	76	Suspect	41	Negative	63	Negative
18	Orbivirus	Warrego	100	Positive	0	negative	16	Positive	95	Positive	5	Positive
19	Orbivirus	Warrego	99	Positive	0.24	negative	7	Positive	96	Positive	4	Positive
20	Orbivirus	Paroo River	100	Positive	-0.18	negative	8	Positive	95	Positive	4	Positive
21	Orbivirus	Corriparta	85	Positive	-0.1	negative	17	Positive	92	Positive	9	Positive
22	Orthobunyavirus	Facey's Paddock	10	Negative	0.09	negative	76	Suspect	43	Negative	42	Negative
23	Orthobunyavirus	Koongol	8	Negative	0.04	negative	90	Negative	47	Negative	56	Negative
24	Orthobunyavirus	Maputta	85	Positive	-0.01	negative	81	Negative	41	Negative	41	Negative
25	Orthobunyavirus	Taggert	65	Positive	0.01	negative	80	suspect	22	Negative	93	Negative
26	Orthobunyavirus	Kawanyama	-17	Negative	0.32	negative	111	Negative	0	Negative	97	Negative
27	Orthobunyavirus	Belmont	-15	Negative	-0.08	negative	102	Negative	62	Positive	46	Negative
28	Ephemerovirus	Bovine Ephemeral Fever	-41	Negative	-0.03	negative	111	Negative	8	Negative	135	Negative
29	Macavirus	Malignant Catarrhal Fever	N/A	N/A	2.33	Positive	118	Negative	5	Negative	139	Negative

Figure S3: Analytical specificity: positive sera to twenty-one orbiviruses and eight other arbovirus positive sera.
